# Etiologies of Infectious Keratitis in Malawi

**DOI:** 10.4269/ajtmh.24-0149

**Published:** 2024-07-16

**Authors:** Khumbo Kalua, Esther S. Misanjo, Thomas M. Lietman, Kevin Ruder, Lina Zhong, Cindi Chen, YuHeng Liu, Danny Yu, Thomas Abraham, Nathaniel Wu, Daisy Yan, Armin Hinterwirth, Thuy Doan, Gerami D. Seitzman

**Affiliations:** ^1^ Department of Ophthalmology, Kamuzu College of Health Sciences, Blantyre, Malawi;; ^2^ Francis I. Proctor Foundation, University of California, San Francisco, California;; ^3^ Department of Ophthalmology, University of California, San Francisco, California

## Abstract

Infectious keratitis is a leading cause of corneal blindness worldwide with little information known about causative etiologies in Malawi, Africa. This area is resource-limited with ophthalmologist and microbiology services. The Department of Ophthalmology at the Kamuzu College of Health Sciences in Blantyre, Malawi, is a participating site of an international corneal ulcer consortium, capriCORN (Comprehensive Analysis of Pathogens, Resistomes, and Inflammatory-markers in the CORNea). In this study, 50 patients with corneal ulcers were swabbed for pathogen identification using RNA-sequencing. Corneal trauma was reported in 41% and 19% of the patients worked in agriculture. A pathogen was identified in 58% of the cases. Fungal pathogens predominated, followed by viruses and bacteria. *Aspergillus*, *Fusarium*, HSV-1, and *Gardnerella* were the most common pathogens detected. 50% of patients reported treatment with an antibiotic before presentation. Pathogens unusual for infectious keratitis, such as *Subramaniula asteroids*, *Aureobasidium pullulans*, and *Gardnerella vaginalis*, were also detected.

## INTRODUCTION

Infectious corneal ulceration remains a leading cause of monocular blindness worldwide.^1^^–^^3^ Little is known about infectious corneal disease in Malawi. With only 12 ophthalmologists serving an entire country,^4^ resource-limited access to microbiology laboratory infrastructure,^5^ and the majority of the population living in rural areas, the burden of corneal ulceration in this area can only be approximated because the leading etiologies of corneal ulceration are simply unknown. A survey of seven districts in southern Malawi approximated corneal scarring to be responsible for 12.3% of the overall blindness burden.^6^ Although corneal scarring can be attributable to many processes, infectious corneal ulceration is a leading cause. Trachoma, a leading cause of infectious corneal blindness, was declared eliminated in Malawi by the WHO in 2022.^7^ This is an excellent example of where a global effort at ophthalmic diagnostics paired with targeted therapeutics allows for effective disease treatment, even within resource-limited areas.^8^ A review of infectious corneal ulcers in neighboring Tanzania suggests that infectious corneal ulcers in this part of the world have a long delay to presentation and are often visually threatening with corneal perforations occurring in 30% and 8% requiring surgical eye removal.^9^ In this northern Tanzanian population, corneal cultures were positive in 50%, with half of the positive cultures resulting filamentous fungi. The rate of HIV positivity in the corneal ulcer population was 16%. Before this review, 99% of all patients admitted for corneal ulcer treatment received antibacterial drops (ciprofloxacin), and 66% received antifungal drops (econazole or natamycin) treatment as initial therapy. After the initiation of a corneal ulcer scraping and microbiology evaluation protocol, 87% of the patients received antifungal drops with antibacterial drops as initial therapy, and all infectious keratitis patients admitted to the hospital were offered HIV counseling. Unlike Tanzania, microbiology surveillance of corneal ulcers has not been explored in Malawi. Local expert opinion, obtained by personal communication, believes most corneal ulcerations in this area to be bacterial with candida more likely in the HIV-positive population. Contact lens wear is uncommon in Malawi. Corneal ulcer scrapings with microbiologic evaluation are not routinely performed in the Department of Ophthalmology in Blantyre, Malawi. Pathogen determination is suggested by clinical examination of the cornea and conjunctival discharge.

The Department of Ophthalmology at the Kamuzu University of Health Sciences in Malawi is one participating site of an international corneal ulcer study (Comprehensive Analysis of Pathogens, Resistomes, and Inflammatory-markers in the CORNea; capriCORN). Here, pathogens of infectious corneal ulcers are determined by metagenomic RNA-sequence analysis (RNA-seq), an unbiased diagnostic approach that allows for the identification of any pathogen in a clinical sample.

## MATERIALS AND METHODS

Patients presenting to the Department of Ophthalmology in Blantyre, Malawi, with corneal infections were recruited to join the capriCORN study. capriCORN was approved by the Institutional Review Boards in Malawi and the University of California San Francisco (UCSF) and adhered to the tenets of the Declaration of Helsinki. All participants underwent swabbing of the cornea for RNA-seq with sterile polyester applicators (Puritan). The swabs were stored in DNA/RNA-Shield (Zymo Research, Irvine, CA) and transferred to a −80°C freezer for storage until shipment to the Proctor Foundation at UCSF for sample processing and analysis. Samples were de-identified, randomized, and processed in the same batch. All laboratory personnel handing samples and interpreting data were masked. RNA-seq library preparation, sequencing, and bioinformatics analyses for pathogen detection had been previously described.^10^^–^^12^ Briefly, 5 µL of extracted RNA of each sample was converted to cDNA and sequencing libraries were prepared using the NEBNext ULTRA II RNA Library Prep Kit for Illumina (New England Biolabs, Ipswich, MA) and sequenced on the NovaSeq 6000 system (Illumina, San Diego, CA) using 150-nucleotide paired-end sequencing. Nonhost reads were aligned to the National Center for Biotechnology nucleotide database. An organism was considered diagnostic if it was known to cause ocular infections and if it represented the most abundant reads in the sample after background water subtraction.

## RESULTS

Fifty patients were swabbed between November 2022 and February 2023, and 39% were female (Table 1). The mean age was 40 (SD ± 17) years. All cases were unilateral. No patients wore contact lenses. The right cornea was involved in 67% (95% CI: 53–79%). Days to presentation was variable with a mean of 49 days (SD ± 121 days) and a median of 7 days (interquartile range: 4–30). Trauma to the cornea with a foreign body was reported in 41% (95% CI: 28–55%). Of these, a metal foreign body was the most reported object in 40% (95% CI: 22–61%). Sixty-five percent (95% CI: 50–76%) of patients presenting for care were already using medicated eye drops. Of these, 46% (95% CI: 33–60%) reported antibacterial eye drops, 4% (95% CI: 0–14%) antifungal eyedrops, 20% (95% CI: 11–33%) steroid eye drops, and 8% (95% CI: 3–19%) did not know the name of their drops. The central cornea was affected in 23% (95% CI: 10–30%) with a hypopyon in 8% (95% CI: 3–20%). Nineteen percent (95% CI: 10–32%) reported working a farming or agriculture profession.

**Table 1:** Demographics of patients with presumed infectious corneal ulcers in Blantyre, Malawi

Characteristic	Total “Yes”/No. Assessed (%)*	95% CI
Sex, %		
Female	39	–
Male	61	–
Age ± SD, years	40 ± 17	–
Duration of Symptoms		
Mean ± SD, days	49 ± 121	–
Median, IQR, days	7 (4–30)	–
Clinical Features*		
Right Eye	33/49 (67)	53–79%
Trauma to Cornea	20/49 (41)	28–55%
Metal Foreign Body	8/20 (40)	22–61%
Agriculture Worker	9/48 (19)	10–32%
Central Cornea Infiltrate	11/48 (23)	13–37%
Hypopyon	4/48 (8)	3–20%
Presented on Drops	31/48 (65)	50–76%
Using Antibacterial Drops	23/50 (46)	33–60%
Using Antifungal Drops	2/50 (4)	0–14%
Using Steroid Drops	10/50 (20)	11–33%

IQR = interquartile range.

* Total “yes”/number assessed (%) unless otherwise noted.

**Figure 1.**
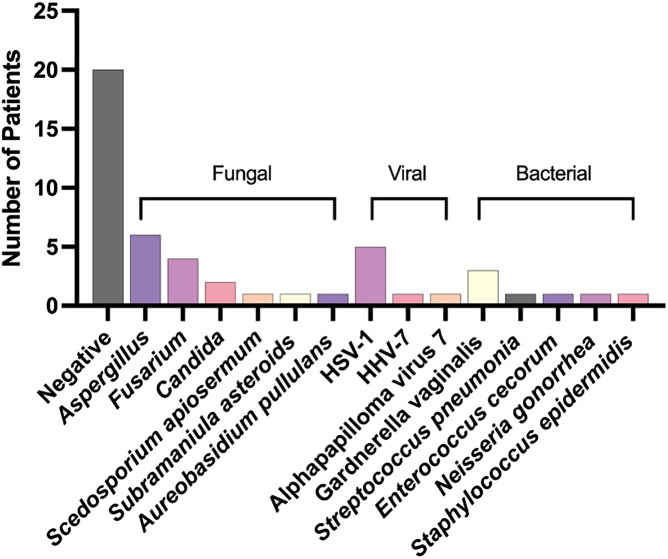
Pathogens identified in corneal ulcers in Blantyre, Malawi.

RNA-seq identified a pathogen in 58% of the corneal ulcers (Figure 1). Here, fungal etiologies were the most prevalent pathogen class identified in 30% of cases. Viruses were the second most common pathogen in 14%, followed by bacterial pathogens in 12%, and a parasite in 2%. Of the fungal pathogens identified, six of 15 (40%) were *Aspergillus *spp., four of 15 (27%) were *Fusarium *spp., and two of 15 (13%) were *Candida *spp. Individual cases of rare fungi were also identified including *Scedosporium apiosermum*, *Subramaniula asteroids*, and *Aureobasidium pullulans*.

Fourteen percent (seven of 50) of the cases were positive for a viral etiology. Human herpes simplex virus 1 (HSV-1) was predominant at 71% (five of seven patients). Human herpes virus 7 (HHV-7) and alphapapillomavirus 7 were identified in the other two patients.

Twelve percent (six of 50) of the cases were positive for a bacterial etiology. Of those, *Gardnerella vaginalis* was detected in 43% (3/6) of the corneal scrapings. *Staphylococcus epidermidis*, *Streptococcus pneumonia*, *Enterococcus cecorum*, and *Neisseria gonorrhea* were also detected.

## CONCLUSION

**Figure 2.**
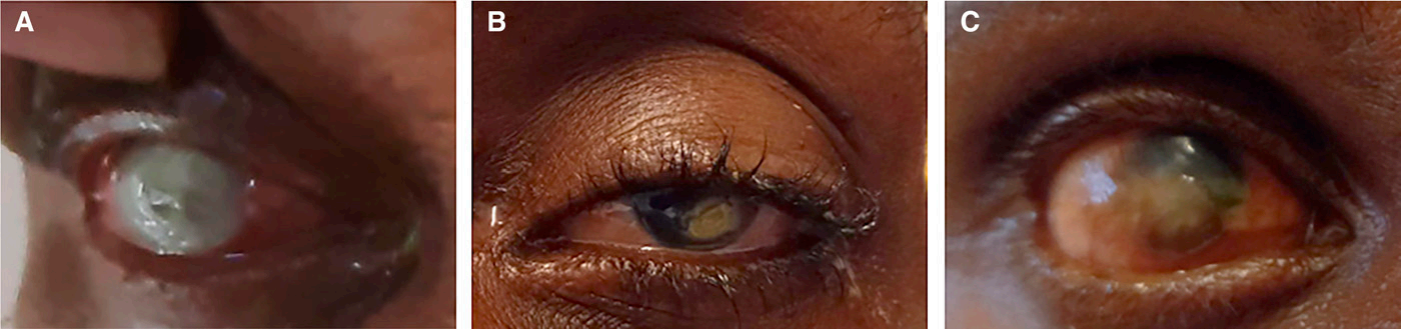
Select clinical examples of corneal ulcers in Blantyre, Malawi (**A**) *Aspergillus flavus*, (**B**) *Subramaniula asteroids*, (**C**) *Alphapapillomavirus* 7.

RNA-seq analysis of corneal ulcers in Blantyre, Malawi, suggests that pathogens responsible for infectious keratitis are varied, with fungal organisms accounting for the majority of pathogen-positive cases. The rate of fungal keratitis in Malawi is similar to that documented in other African countries but is different from what local treating Malawi ophthalmologists (K. K., E. S. M.) suspected clinically.^13^ As is commonly found in tropical climates, filamentous fungi were frequent in this infectious keratitis population.^14^ Although *Aspergillus* species were the most commonly identified fungi (Figure 2A), fungi previously only described in a few case reports were also identified.^15^^–^^17^ *Subramaniula asteroids* (Figure 2B) and *Aureobasidium pullulans* were detected in a 34-year-old female farmer and 44-year-old male welder, respectively.

Bacterial pathogen detection may be decreased because 46% of this infectious keratitis population presents for care while already using topical antibacterial drops. This series is also notable for three cases of keratitis attributable to *Gardnerella vaginalis*. This organism has been reported as causative for conjunctivitis in neonates^18^ but not infectious keratitis. This finding, along with the identification of *Neisseria gonorrhea*–associated keratitis coinfected with one case of *Gardnerella vaginalis*, is likely related to the high rate of sexually transmitted diseases (STDs) in this country.^19^ Local STD prevalence likely also explains the finding of alphapapillomavirus-7 in a corneal ulcer. This virus is a human papilloma virus and is found in high-risk cervical cancers as well as ocular surface tumors.^20^ The patient in whom this virus was identified had both an ocular surface tumor and a corneal ulcer (Figure 2C). Data on the prevalence and etiology of vision loss in Malawi are limited. Access to specialized ophthalmic care including microbiology services is similarly limited. Surveillance of infectious disease etiology is an important first step in allowing for best practice early intervention for patients with corneal ulcers.

This study is limited by sampling at only a single tertiary center in Malawi. In addition, confirmatory microbiologic tests including phenotypic antibiotic resistance results were not locally available. While RNA-seq identified a pathogen in 58% of the corneal ulcers in this series, 42% of the cases did not have a pathogen identified. Potential reasons for the negative results include the following: 1) some ulcers may have been adequately treated before presentation because 50% of the patients presented on antibiotic or antifungal drops, 2) the host immune system may have cleared a causative pathogen to below detectable threshold before scraping, 3) some ulcers may have been noninfectious (e.g., neurotrophic keratitis) or adequately treated, or 4) insufficient biospecimen. Finally, sample collection predominantly occurred during the wet season, and thus the pathogen profile seen in this series may not reflect the pathogen profile in the dry season.

Effective corneal ulcer treatment depends on accurate pathogen identification. However, routine diagnostics for ocular infections generally have low sensitivity due to the inherent limitations of the available tests and/or the low biomass inherent to ocular specimens. Unbiased genome-based testing using high-throughput sequencing can circumvent some of those challenges.^21^ Here, the cataloging of organisms associated with corneal infections in Malawi revealed unexpected pathogens with potential to guide local practice patterns.

## Supplemental Materials

10.4269/ajtmh.24-0149 Supplemental Materials
