# Supplementary material for: Etiologies of Infectious Keratitis in Malawi
Source: Am J Trop Med Hyg. 2024 Jul 16;111(3):694–7. doi: 10.4269/ajtmh.24-0149 (PMC11376150; doi:10.4269/ajtmh.24-0149)
Supplement: Supplemental Materials [file tpmd240149.SD1.pdf]

capriCORN Study Group

Germany

- *Saarland University Medical Center, Homburg* – Berthold Seitz, Elias Flockerzi, Loay Daas, Loïc Hamon, Max Bofferding, Tim Berger, Yaser Abu Dail

Israel

- *Rabin Medical Center, Petah Tikva* – Eitan Livny, Irit Bahar, Lee Goren, Ruti Sella

Malawi

- *Blantyre Institute for Community Outreach, Blantyre* – Esther Misanjo, Hendrix Likongwe, Khumbo Kalua

Mexico

- *Instituto Mexicano de Oftalmología, Santiago de Querétaro, Querétaro* – Carlos Daniel Núñez Amaro, Jaime Macías Martínez, Jose Fernando Pérez Pérez, Van Charles Lansingh

Niger

- *Programme National de Santé Oculaire, Niamey* – Abdou Amza, Abdoul Salam Youssouffou Souley, Abdourahame Bachabi, Adam Nouhou Diori, Adamou Zakari, Aichatou Atto, Bagna Hamidou, Barazé Yacouba, Bonkano Yahaya, Fatouma Sofo, Hadjia Yakoura Abba Kaka, Hassane Amadou Bouba Traore, Ibrahim Mahaman Laouali, Issa Saadou, Kakale Laouali, Lamyne Roufaye, Laouali Laminou, Magagi Mamane Sani, Mahaman Ibrahim, Mariama Boubacar, Mariama Soumana, Mayaki Moctar Yacouba, Mijitaba Hassane, Moctar Issiakou, Moumouni Salifou, Nameywa Boubacar, Ramatou Boulhassane, Sadiaa Abdoulaye, Seley Ali, Yaboubou Soumana, Zakou Abdou

Singapore

- *Singapore National Eye Centre, Singapore* – Hon Shing Ong, Jodhbir S. Mehta, Yu-Chi Liu

South Africa

- *University of the Witwatersrand Charlotte Maxeke Johannesburg Academic Hospital, Johannesburg* – Roland Hollhumer

Switzerland

- *Department of Ophthalmology, Pallas Kliniken, Olten* – Alexandra Bograd, Christoph Tappeiner, David Goldblum

Taiwan

- *School of Medicine, National Yang Min Chiao Tung University, Taipei* – Nai-Wen Fan;

Thailand

- *Chulalongkorn University, Bangkok* – Keeratika Sangsao, Susama Chokesuwattanaskul, Vannarut Satitpitakul;
- *Phramongkutklao College of Medicine, Bangkok* – Wiwan Sansanayudh

United States of America

- *Columbia University Edward S. Harkness Eye Institute, New York, New York* – Andres Serrano, Danielle Trief, Gabriel Rand, George J. Florakis, Janice Kim, Leejee H. Suh;
- *Cornea Associates of Texas, Dallas, Texas* – Joshua Zaffos, Luke Potts;

- *Eyesight Hawaii, Honolulu, Hawaii* – Kristin Hirabayashi;
- *Oregon Health & Science University Casey Eye Institute, Portland, Oregon* – Travis Redd;
- *Price Vision Group* – Anjulie M. Gang, David G. Carli, Francis W. Price Jr., Kathleen N. Dudasko, Marianne O. Price, Matthew T. Feng, Xavier M. Mortensen;
- *University of California, Los Angeles Stein Eye Institute, Los Angeles, California* – Carla Berkowitz, Edmund Tsui, Promporn Patarajierapun, Simon S.M. Fung;
- *University of California, San Diego Shiley Eye Institute, La Jolla, California* – Carol Yu, Esmeralda McClean, Philip Kim;
- *University of California, San Francisco F. I. Proctor Foundation, San Francisco, California* – Armin Hinterwirth, Cindi Chen, Daisy Yan, Danny Yu, Elodie Lebas, Gerami D. Seitzman, Kevin Ouimette, Lina Zhong, Michael S. Deiner, Thomas Abraham, Thomas M. Lietman, Thuy Doan, Travis C. Porco, Yuheng Liu;
- *University of Utah Moran Eye Center, Salt Lake City, Utah* – Amy Lin, Brian Zaugg, Elizabeth Nuttall, Karen Gutierrez, Katherine S. Hu;
- *University of Wisconsin School of Medicine and Public Health, Madison, Wisconsin* – Kevin Kurt, Sarah Nehls;
- *Washington University in St. Louis, St. Louis, Missouri* – Jennifer Enright, Jessica Walia, Praneetha Thulasi;
- *Program Officer*: George A. McKie
